# Vying for the control of inflammasomes: The cytosolic frontier of enteric bacterial pathogen–host interactions

**DOI:** 10.1111/cmi.13184

**Published:** 2020-03-17

**Authors:** Julia Sanchez‐Garrido, Sabrina L. Slater, Abigail Clements, Avinash R. Shenoy, Gad Frankel

**Affiliations:** ^1^ Department of Life Sciences Imperial College London London UK; ^2^ Department of Infectious Disease, MRC Centre for Molecular Bacteriology & Infection Imperial College London London UK

**Keywords:** *E. coli*, inflammasomes, *Listeria monocytogenes*, *Salmonella*, *Shigella*, *Yersinia*

## Abstract

Enteric pathogen–host interactions occur at multiple interfaces, including the intestinal epithelium and deeper organs of the immune system. Microbial ligands and activities are detected by host sensors that elicit a range of immune responses. Membrane‐bound toll‐like receptors and cytosolic inflammasome pathways are key signal transducers that trigger the production of pro‐inflammatory molecules, such as cytokines and chemokines, and regulate cell death in response to infection. In recent years, the inflammasomes have emerged as a key frontier in the tussle between bacterial pathogens and the host. Inflammasomes are complexes that activate caspase‐1 and are regulated by related caspases, such as caspase‐11, ‐4, ‐5 and ‐8. Importantly, enteric bacterial pathogens can actively engage or evade inflammasome signalling systems. Extracellular, vacuolar and cytosolic bacteria have developed divergent strategies to subvert inflammasomes. While some pathogens take advantage of inflammasome activation (e.g. *Listeria monocytogenes, Helicobacter pylori*), others (e.g. *E. coli*, *Salmonella*, *Shigella, Yersinia* sp.) deploy a range of virulence factors, mainly type 3 secretion system effectors, that subvert or inhibit inflammasomes. In this review we focus on inflammasome pathways and their immune functions, and discuss how enteric bacterial pathogens interact with them. These studies have not only shed light on inflammasome‐mediated immunity, but also the exciting area of mammalian cytosolic immune surveillance.

**Abbreviations**
A/E lesion: attachment and effacement lesion
ALRs: AIM2 (absent in melanoma 2)‐like receptors
ASC: apoptotic speck associated protein with a CARD
BIR: baculovirus inhibitor of apoptosis protein repeat
CARD: caspase activation and recruitment domain
DC: dendritic cell
GBPs: guanylate binding proteins
GSDMD: gasdermin D
LEE: locus for enterocyte effacement
NAIP: nucleotide‐binding domain and leucine rich repeat containing (NLR)‐containing apoptosis‐inhibitory protein
NF‐κB: nuclear factor kappa‐light‐chain‐enhancer of activated B cells
NLR: nucleotide‐binding domain and leucine rich repeat containing (NLR)
NLRCs: nucleotide‐binding domain and leucine rich repeat containing (NLR) family CARD domain‐containing proteins
NLRPs: nucleotide‐binding domain and leucine rich repeat containing (NLR) family PYD domain‐containing proteins
SPI: *Salmonella* pathogenicity island
T3SS: type 3 secretion system
TLRs: toll‐like receptors
TRIM: tripartite motif

## 1 INTRODUCTION

Acute gastroenteritis is caused by infection of the stomach or intestinal mucosa with enteric pathogens, which are typically transmitted via contaminated food or water. It is characterised by damage to the mucosa and loss of mucosal barrier integrity, leading to malabsorption, diarrhoea and consequent dehydration (DuPont, 2009). Infectious gastroenteritis can affect people of all ages, thus having a substantial economic impact worldwide. In the USA, 179 million people suffer from acute gastroenteritis per year, leading to over 5,000 deaths (DuPont, 2009; Shane et al., 2017); in the UK the estimate is of 17 million cases per year (Tam et al., 2012). The most common causes of bacterial intestinal infections include the Gram‐negative pathogens *Salmonella enterica*, *Shigella* sp., *Escherichia coli* and *Yersinia* and the Gram‐positive pathogen *Listeria monocytogenes* (Kirk et al., 2015; Shane et al., 2017). While its mode of transmission is not currently established, *Helicobacter pylori* is the most prevalent aetiological agent of bacterial gastritis and is a major risk factor for the development of gastric malignancies (Chey, Leontiadis, Howden, & Moss, 2017).

The gastrointestinal immune system, which encompasses both immune and intestinal epithelial cells (IECs) lining the mucosa, must recognise and be activated by pathogenic insults, while remaining anergic to the presence of the endogenous microbiota. One of the mechanisms involved in this distinction is the multiprotein cytosolic complex known as the inflammasome. The inflammasome acts as a molecular platform for caspase‐1 activation and has been shown to have an increasingly important role in innate immunity since it was described in 2002 (Martinon, Burns, & Tschopp, 2002). It assembles in response to microbial or danger signals, triggering downstream signalling cascades that give rise to the release of pro‐inflammatory factors, including cytokines (e.g. interleukin‐1β [IL‐1β] and IL‐18) and alarmins (such as IL‐1α and HMGB1), as well as pyroptotic cell death (Broz & Dixit, 2016; Hayward, Mathur, Ngo, & Man, 2018). In the intestine, inflammasome signalling is functional within myeloid cells, such as macrophages, dendritic cells (DCs) and neutrophils, as well as epithelial cells, pointing to their pivotal role in the early response to pathogens.

## 2 THE INFLAMMASOME COMPONENTS

**Figure 1:**
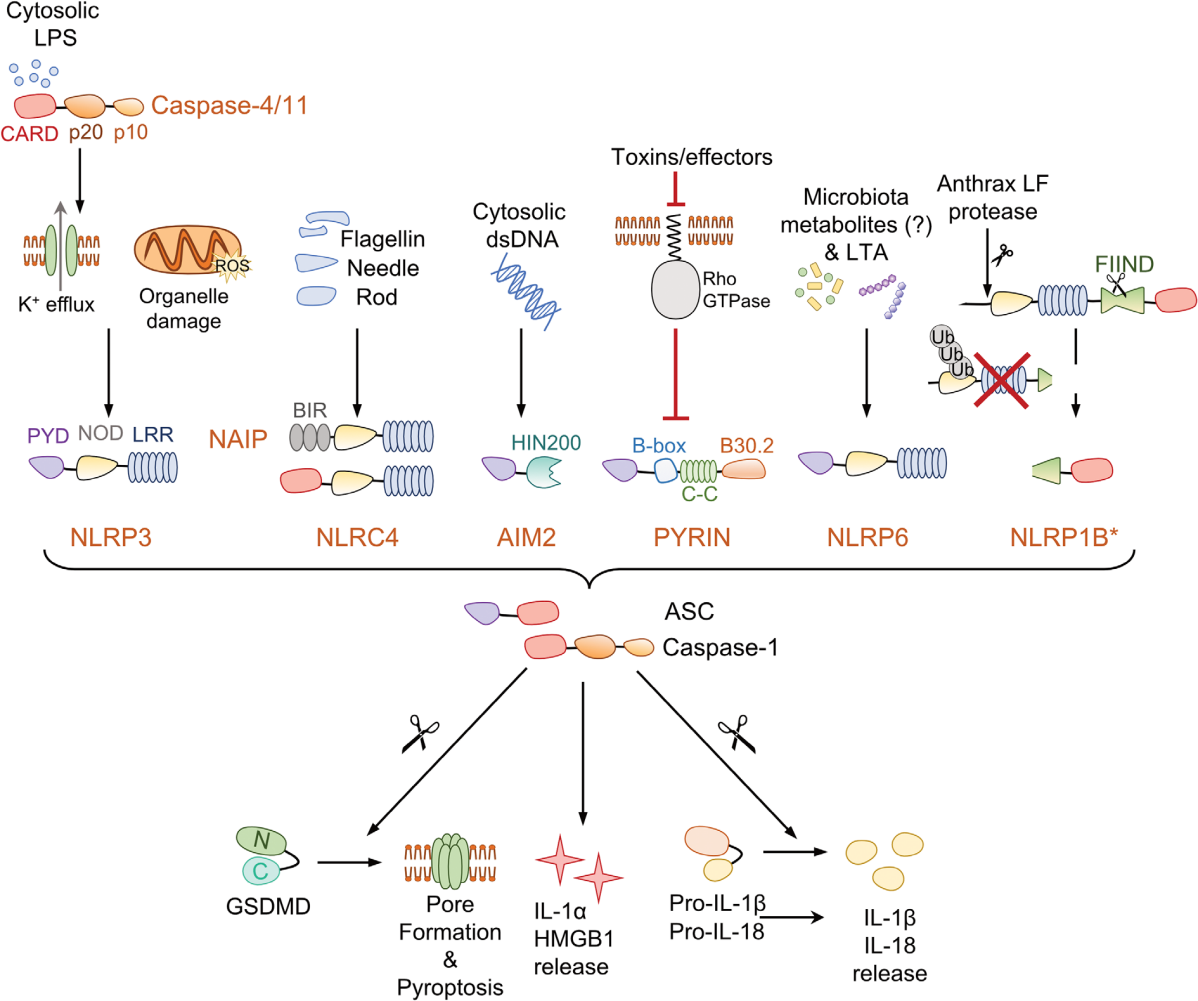
Inflammasome‐forming sensors and their known activators. Inflammasomes are multiprotein complexes that function as platforms to activate caspase‐1. Some inflammasome sensors, such as NLRP3, PYRIN and NLRP1B, are activated following perturbations of cellular homeostasis triggered by damage or microbial associated molecular patterns. For example, mitochondrial or lysosomal disruption will lead to NLRP3 activation, while inhibition of host Rho‐GTPases will allow PYRIN inflammasome assembly and degradation of the NLRP1B N‐terminal will lead to nucleation of the free CARD‐containing NLRP1B C‐terminus. Other inflammasome sensors, exemplified by AIM2, NAIP‐NLRC4 and caspase‐11 (caspase‐4 and 5 in humans), are activated in response to direct detection of their ligands: DNA is recognised by the AIM2 HIN200 domain, NAIP proteins bind flagellin and type 3 secretion system (T3SS) needles and rods, and the caspase‐11 CARD domain interacts with LPS. Active caspase‐11/4/5 cleaves Gasdermin D (GSDMD), leading to pore formation and subsequent potassium efflux, which can trigger non‐canonical activation of the NLRP3 inflammasome, and pyroptosis. NLRP6 functions as a direct sensor of lipoteichoic acid (LTA), but can also be activated by changes in the microbiota and has additionally been shown to perform inflammasome‐independent functions. *Human NLRP1 has an N‐terminal PYD domain. Domain compositions are colour coded and abbreviated as follows: CARD, caspase‐activation and recruitment domain; p20 and p10, large and small catalytic subunits; PYD, pyrin domain; NOD, nucleotide binding and oligomerisation domain; LRR, leucine rich repeat; BIR, baculovirus inhibitor of apoptosis domain; HIN200, haematopoietic expression, interferon inducible, nuclear localised (HIN) DNA binding domain of ∼200 residues; C‐C, coiled‐coil; FIIND, function to find domain

The assembly of inflammasomes is triggered by the recognition of a signal by a cytosolic sensor, which can be a member of the NLR family (e.g. NLRP3 or NLRC4), an ALR, or the TRIM protein PYRIN (Broz & Dixit, 2016) (Figure 1). The NLRs are further divided based on their N‐terminal protein–protein interaction domains, for example NAIPs (also known as NLRBs) contain BIR, NLRCs contain CARD and NLRPs contain pyrin motifs (except NLRP1, which contains a CARD). The pyrin domain is also present in ALRs and PYRIN. The pyrin domain mediates interactions with the adaptor ASC, a small protein that itself consists of a pyrin domain and a CARD, which promotes the recruitment of pro‐caspase‐1 to oligomerised inflammasomes. This leads to caspase‐1 oligomerisation and proximity‐induced activation via autoproteolysis (Figure 1). While non‐CARD‐containing sensors require ASC to recruit pro‐caspase‐1, NLRCs can interact via CARD and directly activate full‐length pro‐caspase‐1. This leads to ASC‐independent pyroptosis, but ASC is still required for caspase‐1 autoproteolysis and cytokine processing (Broz, von Moltke, Jones, Vance, & Monack, 2010). NAIPs can only activate inflammasomes by stimulating NLRC4, which in turn activates pro‐caspase‐1. Proteolytically activated caspase‐1 cleaves pro‐IL‐1β and pro‐IL‐18 into their bioactive forms.

Caspase‐mediated cleavage of GSDMD liberates its N‐terminal fragment that oligomerises, binds to lipids (e.g. phosphatidylinositol phosphates, phosphatidylserine and cardiolipin) and forms large pores in the plasma membrane and mitochondria (Broz, Pelegrín, & Shao, 2019; Ding et al., 2016; Liu et al., 2016). GSDMD pores can then facilitate the release of mature cytokines and eventually lead to osmotic swelling and a form of lytic cell death called pyroptosis (Kayagaki et al., 2015; Shi et al., 2015; Evavold et al., 2018; Orning, Lien, & Fitzgerald, 2019). Not all cells with active inflammasomes undergo pyroptosis, as the endosomal sorting complexes required for transport machinery can promote repair of GSDMD‐mediate plasma membrane damage, promoting cellular survival and reducing cytokine release (Rühl et al., 2018). Notably, pyroptosis‐independent release of inflammasome‐derived cytokines has also been shown, for example from monocytes exposed to LPS (Gaidt et al., 2016) and neutrophils infected with *Salmonella* (Chen et al., 2014). Other mechanisms of cytokine release during inflammasome activation have been proposed, including autophagy‐associated unconventional secretion (Dupont et al., 2011; Kimura et al., 2017), exosome release from multivesicular bodies (MacKenzie et al., 2001; Qu, Franchi, Nunez, & Dubyak, 2007) and passive release after cell lysis (Cullen, Kearney, Clancy, & Martin, 2015).

## 3 SIGNALS FOR INFLAMMASOME ACTIVATION

It is commonly accepted that inflammasome activation in myeloid cells and the resultant IL‐1β release require two signals. The ‘priming’ signals (signal 1) activate the transcription and/or post‐translational regulation of inflammasome‐associated genes/proteins and are best understood for the activation of the NLRP3 inflammasome in macrophages. These include: (i) the transcription of pro‐IL‐1β, (ii) the transcriptional and translational licensing of NLRP3 via toll‐like receptors (TLRs) and NF‐κB (Bauernfeind et al., 2009; Fernandes‐Alnemri et al., 2013; Lin et al., 2014), (iii) interferon‐dependent up‐regulation of murine caspase‐11 (Rathinam et al., 2012; Benaoudia et al., 2019) and mouse and human GBPs (Kim et al., 2016) and (iv) IRF2‐driven expression of human caspase‐4 (Benaoudia et al., 2019) and mouse GSDMD (Kayagaki et al., 2019). Importantly, bacterial ligands such as cell wall components and nucleic acids can serve as signal 1, and pathogen‐associated virulence factors and/or activities, as discussed below, serve as a second signal specific to the NLR/ALR/PYRIN inflammasome sensors. Thus, during infection by bacterial pathogens, inflammasome signalling is a fast, inflammatory process that cooperates with other innate immune signalling pathways such as TLRs and interferons.

Several bacterial molecules can serve as signal 2 during activation of NAIPs (Figure 1) (Hayward et al., 2018; Evavold & Kagan, 2019). Bacterial flagellin and evolutionarily conserved rod and needle proteins of the type 3 secretion system (T3SS) injectisome of some Gram‐negative bacteria are recognised by the NAIPs, leading to the assembly of the NLRC4 inflammasome in macrophages (Miao, Mao, et al., 2010; Zhao et al., 2011). There are seven mouse *Naip* genes, four of which recognise different ligands (Kofoed & Vance, 2011; Zhao et al., 2016), while humans only encode one *NAIP* gene that can recognise a similar repertoire of ligands (Yang, Zhao, Shi, & Shao, 2013; Kortmann, Brubaker, & Monack, 2015; Grandjean et al., 2017; Reyes Ruiz et al., 2017).

Cytosolic double‐stranded DNA (dsDNA) is recognised by AIM2 via its HIN domain (Hornung et al., 2009). While PYRIN is autoinhibited through interactions with 14‐3‐3 proteins, it can be activated upon the inactivation of cellular RhoA GTPases by various bacterial toxins (Xu et al., 2014; Masters et al., 2016). The mouse NLRP1B sensor is also basally autoinhibited through its N‐terminal regions, which can undergo proteasomal degradation, for example in response to anthrax lethal toxin, leading to the release of the CARD‐containing C‐terminal fragment that oligomerises to recruit caspase‐1 and activate the NLRP1 inflammasome (Chui et al., 2019; Sandstrom et al., 2019; Xu et al., 2019). Other than the inhibitors of the serine proteases DPP8/9 (Okondo et al., 2017; Zhong et al., 2018), physiological activators of human NLRP1 inflammasome remain to be discovered.

The NLRP3 inflammasome responds to the widest range of stimuli, including potassium efflux, extracellular ATP, increased intracellular calcium, mitochondrial DNA and reactive oxygen species (ROS) and liberation of cathepsins from the lysosome (Swanson, Deng, & Ting, 2019). During infection by Gram‐negative bacteria, the NLRP3 inflammasome can be indirectly activated by murine caspase‐11 (represented by caspase‐4 and 5 in humans), through what is now known as the ‘non‐canonical’ inflammasome signalling pathway. In this pathway, cytosolic LPS is recognised by caspase‐4/5 or caspase‐11, which oligomerises and becomes active (Kayagaki et al., 2013; Shi et al., 2014). Activated caspase‐4/5 or caspase‐11 cleaves GSDMD, leading to pyroptosis and/or to potassium efflux and activation of the NLRP3 inflammasome in macrophages (Kayagaki et al., 2015; Shi et al., 2015). Activation of caspase‐11 has also been linked to modulation of the actin cytoskeleton, phagosome maturation and leukocyte migration (Li et al., 2007; Akhter et al., 2012), showing that this caspase also has non‐inflammatory roles.

Other less well‐studied NLRs include NLRP6, NLRP12, NLRC3 and NLRC5. In particular, NLRP6 is important in the gastrointestinal tract as it is expressed in myeloid cells, IECs and goblet cells, where it contributes to intestinal homeostasis (Elinav et al., 2011; Wlodarska et al., 2014). Notably, NLRC3, NLRP6 and NLRP12 are implicated in the suppression of the inflammatory response, and deficiency of these proteins can confer resistance to infection *in vivo* (Anand et al., 2012; Zaki, Man, Vogel, Lamkanfi, & Kanneganti, 2014; Zhang et al., 2014).

Inflammasomes play an important role in innate immunity, where IL‐1β, IL‐18 and alarmins (e.g. IL‐1α and HMGB1) help promote inflammation, neutrophil recruitment and an adequate adaptive immune response (Mantovani, Dinarello, Molgora, & Garlanda, 2019), while pyroptosis eliminates intracellular bacterial replicative niches (Miao, Leaf, et al., 2010). The antibacterial function of inflammasomes is illustrated by the ability of various inflammasome sensor proteins to recognise and respond to bacterial ligands (Eldridge & Shenoy, 2015; Hayward et al., 2018), and the increased susceptibility to infection shown by mice with deletions in inflammasome components (Man, Karki, & Kanneganti, 2017; Lacey & Miao, 2019). Although inflammasomes have been best studied in myeloid cells, several inflammasome components, such as caspase‐4/11 and pro‐IL‐18, are also expressed in IECs (Man, 2018; Winsor, Krustev, Bruce, Philpott, & Girardin, 2019). The constitutive expression of caspase‐4/11 and NAIPs in IECs (Knodler et al., 2014; Sellin et al., 2014) might allow rapid sensing of Gram‐negative pathogens at mucosal sites. The importance of inflammasomes in innate immune signalling in the gut in response to microbial and sterile insults, as well as the contribution of inflammasomes to diseases such as IBD, cancer and obesity and their role in the maintenance of the intestinal microbiota have been reviewed elsewhere (Levy, Kolodziejczyk, Thaiss, & Elinav, 2017; Man, 2018; Winsor et al., 2019). This review focuses on the host's detection of enteric pathogens via inflammasomes, and the mechanisms used by these pathogens to subvert inflammasome functions to promote their survival and sustain infection.

## 4 SUBVERSION OF INFLAMMASOMES BY ATTACHING AND EFFACING (A/E) PATHOGENS

Bacteria that cause attaching and effacing (A/E) lesions include the diarrhoeagenic human pathogens enterohaemorrhagic *Escherichia coli* (EHEC) and enteropathogenic *E. coli* (EPEC) and the rodent pathogens *Citrobacter rodentium* and rabbit EPEC (Frankel et al., 1998). While EPEC mainly affects young children in low‐ and middle‐income countries (Hu & Torres, 2015), EHEC infection is prevalent in industrial countries and is associated with haemorrhagic colitis and hemolytic uremic syndrome (HUS) (Kampmeier, Berger, Mellmann, Karch, & Berger, 2018).

A/E lesions are characterised by effacement of the brush border microvilli and intimate bacterial attachment to the apical membrane of IECs. Intimate attachment is mediated by strong interactions between intimin on the bacterial surface and Tir (translocated intimin receptor), which is injected into IECs by the T3SS (Frankel & Phillips, 2008; Slater, Sågfors, Pollard, Ruano‐Gallego, & Frankel, 2018), all of which are encoded within the locus for enterocyte effacement (LEE) common to A/E pathogens (Frankel et al., 1998). Intimin:Tir interactions lead to Tir clustering and actin polymerisation underneath attached bacteria, which facilitates delivery of other T3SS effectors that subvert mammalian cell processes (Shenoy, Furniss, Goddard, & Clements, 2018). Strain to strain variability in both the repertoire of T3SS effectors and other virulence factors affects the outcome of pathogen–host interactions; haemolysins (such as EhxA) and the Shiga toxins (Stx1 and 2) mentioned herein are exclusively expressed by EHEC.

**Figure 2:**
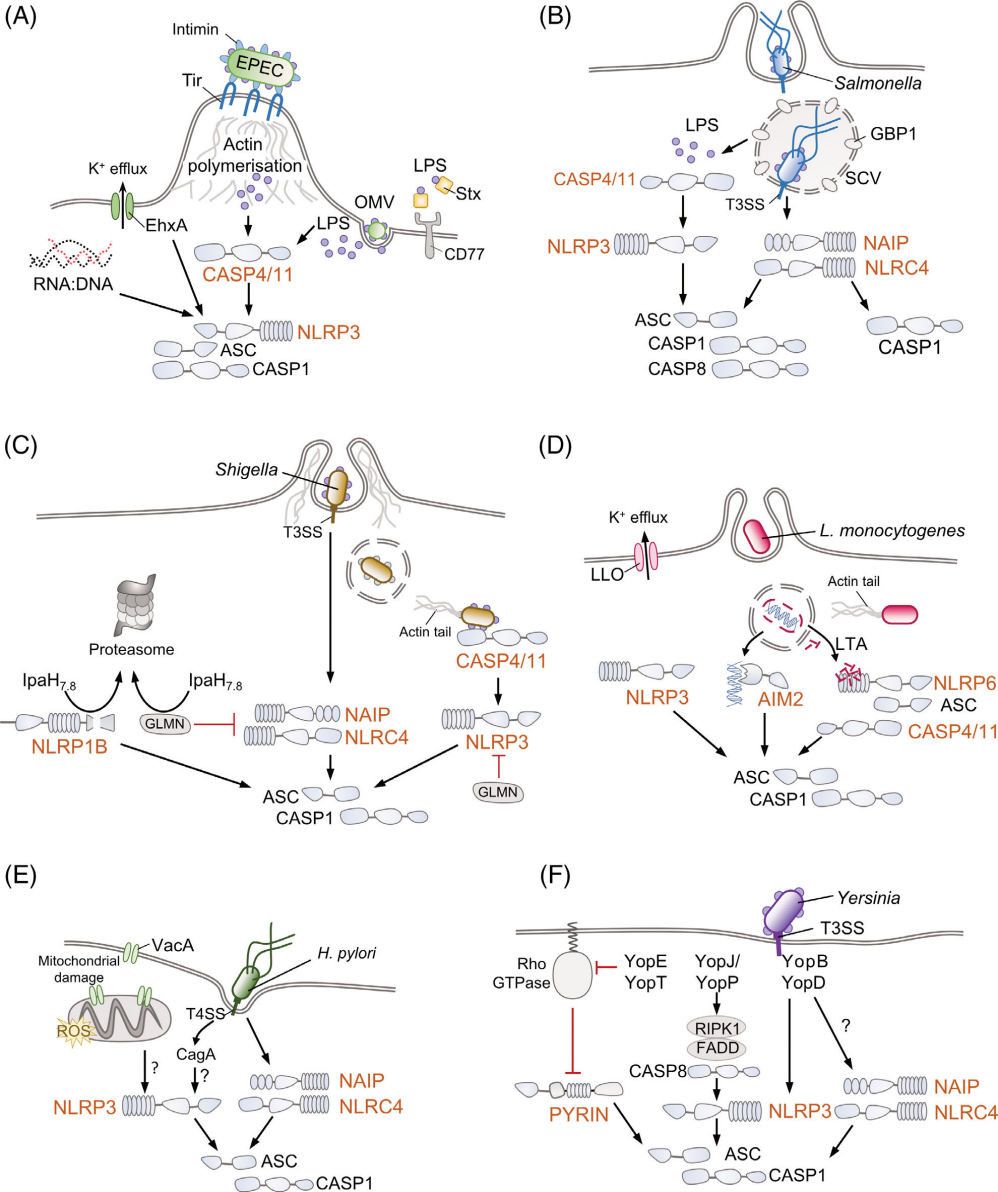
Enteric pathogens can activate multiple inflammasome pathways. Schematics from (a) to (f) show how various enteric pathogens stimulate the assembly and activation of different inflammasomes, focussing on the activating signal (signal 2). Downstream consequences of inflammasome activation shown in Figure 1, that is Gasdermin D cleavage, pore formation and pyroptosis, caspase‐1‐mediated cleavage of pro‐IL‐1β and pro‐IL‐18 into their active forms and the release of these pro‐inflammatory cytokines together with alarmins, are not depicted for simplicity. A/E pathogens (such as Enteropathogenic and Enterohaemorrhagic *E. coli*, EPEC and EHEC) (a), *Helicobacter pylori* (e) and *Yersinia* (f) are mainly extracellular pathogens, while *Salmonella* survives intracellularly in *Salmonella* containing vacuoles (SCVs) (b) and *Shigella* (c) and *Listeria monocytogenes* (d) are cytosolic bacteria that escape the vacuoles and can move from cell to cell via manipulation of the host actin cytoskeleton. Host proteins are featured in greyscale to emphasise the role of bacterial factors. See text for further information on the mechanisms employed by these pathogens to evade or subvert inflammasomes. OMV, outer membrane vesicle; Stx, Shiga toxin; Tir, translocated intimin receptor; GBP1, guanylate binding protein 1; GLMN, glomulin; LLO, listeriolysin; LTA, lipoteichoic acid; VacA, vacuolating cytotoxin A; CagA, cytotoxin‐associated gene A; ROS, reactive oxygen species; KD, kinase domain; RHIM, RIP (receptor‐interacting serine/threonine‐protein) homotypic interaction motif; DD, death domain; Yop, Yersinia outer protein

While the multiplicity of virulence mechanisms employed by A/E pathogens underpins their success, it also amplifies the host's ability to sense and respond to their threat. Activation of the NLRP3 inflammasome by EPEC‐ and EHEC‐derived signals is well documented. For example, the EHEC haemolysin EhxA can cause membrane pores, potassium efflux and NLRP3‐caspase‐1 activation in human macrophages (Cheng et al., 2015). Stx also induces pyroptosis in macrophages via canonical NLRP3 activation and IL‐1β release, in addition to apoptosis via caspase‐3/8 activation (Lee et al., 2016). Like other AB_5_ toxins of its kind (e.g. cholera toxin) (Kayagaki et al., 2013), Stx alone can activate the inflammasome by facilitating the delivery of LPS to the macrophage cytosol, leading to activation of caspase‐4, mitochondrial ROS production and subsequent non‐canonical activation of the NLRP3 inflammasome (Platnich et al., 2018). In contrast, Vanaja et al. found only marginal NLRP3 induction by EhxA or Stx, but identified RNA:DNA hybrids as the microbial molecules that stimulate NLRP3 in murine macrophages (Vanaja et al., 2014). The different findings in these studies could be explained by different bacterial growth conditions, which can affect the levels of EhxA or Stx expression, or the use of macrophages of human vs. mouse origin. Similar responses involving mouse caspase‐11 and NLRP3 were observed with enterotoxigenic *E. coli* and non‐pathogenic *E. coli* strains, suggesting this is a broad host‐driven response to cytosolic bacterial LPS (Figure 2a; Table 1).

**Table 1:** Enteric pathogens and the inflammasomes: activation and inhibition

Pathogen	Inflammasome	Virulence factor	Effect	References
**A/E pathogens**	CASP4/Casp11	LPS (T3SS‐independent) (EPEC/EHEC)	Activator	(Knodler et al., 2014; Vanaja et al., 2016; Goddard et al., 2019)
	CASP4 → NLRP3	Stx (EHEC)		(Lee et al., 2016; Platnich et al., 2018)
		Tir and LPS (T3SS‐dependent) (EPEC)		(Goddard et al., 2019)
	NLRP3	Haemolysin EhxA (EHEC)		(Zhang et al., 2012; Cheng et al., 2015)
	Nlrp3	RNA:DNA (EHEC)		(Vanaja et al., 2014)
	Naip2‐Nlrc4	EscI (T3SS rod; EPEC/EHEC), EprJ (ETT2 rod; EHEC)		(Miao, Mao, et al., 2010; Zhao et al., 2011; Wu et al., 2019)
	NAIP‐NLRC4 **OR** Naip1‐Nlrc4	EprI (ETT2 needle; EHEC)		(Yang et al., 2013; Zhao et al., 2016; Wu et al., 2019)
	NLRP3	NleA/EspI (EPEC)	Inhibitor	(Yen et al., 2015)
	CASP4/Casp11	NleF (EPEC)		(Blasche et al., 2013; Pallett et al., 2017; Pollock et al., 2017)
***Salmonella* Typhimurium**	NAIP‐NLRC4 **OR** Naip5/6‐Nlrc4	Flagellin	Activator	(Broz, Newton, et al., 2010; Miao, Leaf, et al., 2010; Kofoed & Vance, 2011; Zhao et al., 2011; Franchi et al., 2012; Kortmann et al., 2015; Reyes Ruiz et al., 2017)
	NAIP‐NLRC4 **OR** Naip2‐Nlrc4	PrgJ (inner SPI‐1 T3SS rod)		(Kofoed & Vance, 2011; Zhao et al., 2011; Franchi et al., 2012; Tenthorey, Kofoed, Daugherty, Malik, & Vance, 2014; Reyes Ruiz et al., 2017)
	NAIP‐NLRC4 **OR** Naip1‐Nlrc4	PrgI (SPI‐1 T3SS needle)		(Zhao et al., 2011; Yang et al., 2013)
	CASP4/Casp11 → NLRP3/Nlrp3	LPS		(Shenoy et al., 2012; Meunier et al., 2014; Fisch et al., 2019)
	CASP4/Casp11	LPS		(Knodler et al., 2010; Broz et al., 2012; Knodler et al., 2014)
	Nlrp3	Curli fibres		(Rapsinski et al., 2015)
		? (↓NADH, ↑mitoROS)*		(Sanman et al., 2016)
	Nlrp3?*	SlrP	Inhibitor	(Rao et al., 2017)
	Casp11 → Nlrp3	Aconitase		(Wynosky‐Dolfi et al., 2014)
***Shigella***	CASP4/Casp11	LPS	Activator	(Kobayashi et al., 2013; Shi et al., 2014; Watson et al., 2019)
	Naip2‐Nlrc4	MxiI (T3SS rod)		(Miao, Mao, et al., 2010; Yang et al., 2013; Suzuki, Franchi, et al., 2014; Zhao et al., 2016)
	Naip‐NLRC4 **OR** Naip1‐Nlrc4	MxiH (T3SS needle)		(Yang et al., 2013; Zhao et al., 2016)
	NLRC4/Nlrc4?	IpaB		(Senerovic et al., 2012)
	NLRP3/Nlrp3 & NLRC4/Nlrc4	IpaH7.8		(Suzuki, Mimuro, et al., 2014; Suzuki et al., 2018)
	Nlrp1b	IpaH7.8		(Neiman‐Zenevich et al., 2017; Sandstrom et al., 2019)
	CASP4	OspC3	Inhibitor	(Kobayashi et al., 2013)
***Listeria monocytogenes***	NLRP3/Nlrp3	Listeriolysin (LLO)	Activator	(Zwaferink, Stockinger, Hazemi, Lemmens‐Gruber, & Decker, 2008; Meixenberger et al., 2010; Sauer et al., 2011; Eldridge et al., 2017)
	Nlrp3	p60		(Schmidt & Lenz, 2012)
		RNA		(Kanneganti et al., 2006)
	Aim2	dsDNA		(Kim et al., 2010; Rathinam et al., 2010; Tsuchiya et al., 2010; Warren et al., 2010; Wu et al., 2010)
	Naip‐Nlrc4	Flagellin		(Warren, Mao, Rodriguez, Miao, & Aderem, 2008; Sauer et al., 2010; Tsuchiya et al., 2010; Warren et al., 2010)
	Nlrp6‐Casp4	Lipoteichoic acid (LTA)		(Meixenberger et al., 2010; Hara et al., 2018)
	CASP4	?*		(Hara et al., 2018)
	Nlrp1b	Host cell energy stress?*		(Neiman‐Zenevich et al., 2017)
***Yersinia***	Nlrc4?* (in vivo)	YopB, YopD (*Yps*)	Activator	(Brodsky et al., 2010)
	Nlrp3	YopB, YopD (*Yps*)		(Brodsky et al., 2010; Zwack et al., 2015)
	CASP4/Casp11	LPS (*Yps*)		(Casson et al., 2013; Casson et al., 2015)
	Rip1‐Casp8‐Casp1	YopJ (*Yps*)		(Philip et al., 2014; Weng et al., 2014)
	Pyrin	YopE, YopT (*Yps*)		(Chung et al., 2016; Ratner et al., 2016)
	Naip5‐Nlrc4	Flagellin (*Ye*)		(Matusiak et al., 2015)
	Nlrp3	YopK (*Yps*)	Inhibitor	(Brodsky et al., 2010, Zwack et al., 2015)
	Pyrin/ Casp1	YopM (*Yps*)		(LaRock & Cookson, 2012; Chung et al., 2014; Chung et al., 2016; Ratner et al., 2016)
	NLRP3/Nlrp3	YopH, YopE (*Ye*)		(Schotte et al., 2004; Thinwa et al., 2014)

*Notes*: Bacterial components and infection‐mediated alterations of host cells that lead to inflammasome activation are shaded in orange; inflammasome inhibitors are shown shaded in blue. Indirect inhibitors of NLRP3, for example via inhibition of NF‐κB signalling, are not featured in the table (refer to text). *H. pylori*‐encoded direct activators/inhibitors of inflammasome activation not well defined, and thus the pathogen is not included. When the human inflammasome genes have been involved, these are shown in uppercase (e.g. NLRP3); mouse genes are shown in lowercase except the first letter (e.g. Nlrp3). * indicates when the study does not define which inflammasome is involved or what the bacterial activating signal/factor is.

Abbreviations: mitoROS, mitochondrial reactive oxygen species; *Ye*, *Yersinia enterocolitica*; *Yps*, *Yersinia pseudotuberculosis*.

Recently, EPEC expressing the T3SS has been shown to activate caspase‐4 and NLRP3 in human macrophages. Induction of cell death was dependent on Tir‐mediated actin polymerisation and binding of LPS to caspase‐4, leading to activation of an atypical, non‐canonical signalling pathway via GSDMD to stimulate the NLRP3 inflammasome (Goddard et al., 2019). Surprisingly, EPEC‐induced caspase‐4 activation was not sufficient to induce pyroptosis; induction of pyroptosis, as well as cytokine processing, required the NLRP3‐ASC‐caspase‐1 inflammasome. LPS stimulation by non‐pathogenic *E. coli* (including EPEC grown in conditions that do not stimulate virulence gene expression) takes 16–18 h to evoke a non‐canonical inflammasome response (Vanaja et al., 2016), but the translocation of a functional Tir drives non‐canonical NLRP3 inflammasome activation and IL‐1β release within 4 h (Goddard et al., 2019). It is plausible that Tir‐induced actin polymerisation during EPEC infection facilitates entry of LPS and activation of the atypical inflammasome signalling pathway. Notably, EHEC Tir, which alone does not induce actin polymerisation, can only trigger pyroptotic signalling when provided with its cognate actin polymerising effector TccP (Garmendia et al., 2004; Goddard et al., 2019). Consistent with a role for Tir‐driven actin polymerisation in inflammasome activation, over‐expression of EspJ, an ADP ribosyl transferase (Young et al., 2014) targeting multiple non‐receptor tyrosine kinases (Pollard et al., 2018) that phosphorylate and activate EPEC Tir (Swimm et al., 2004), inhibits EPEC‐induced cell death (Goddard et al., 2019) (Figure 2a). Future studies are needed to investigate the interplay between the cytoskeleton and caspase‐4/11, which has been shown to modulate actin polymerisation at the leading edge (Li, Yin, & Yuan, 2008) and cell migration (Li et al., 2007).

In addition to T3SS effectors, structural T3SS components also provoke inflammasome activation. The inner rod protein EscI of the LEE‐encoded T3SS, the inner rod protein EprJ and the needle protein EprI of the *E. coli* type III secretion system 2 (ETT2) activate murine NLRC4 inflammasomes *in vitro* when introduced directly into the macrophage cytosol (Miao, Mao, et al., 2010; Zhao et al., 2011; Yang et al., 2013; Wu et al., 2019); however, the ETT2 has accumulated considerable mutational attrition and is not believed to form a functional T3SS (Ren et al., 2004). Critically, the EPEC T3SS needle protein EscF is one of the needle proteins that are not detected in human THP‐1 macrophages (Yang et al., 2013). Similarly, the EPEC/EHEC flagellin (FliC) is not recognised by murine NAIP5/human NAIP (Zhao et al., 2011), whereas its *E. coli* K12 counterpart is readily detected (Yang et al., 2014). Taken together, murine NLRC4 may detect EPEC/EHEC expressing the LEE T3SS however, these bacteria likely evade this inflammasome in human macrophages.

Inflammasomes also contribute to A/E pathogen–host interactions *in vivo*. These studies were performed using the extracellular mouse‐specific pathogen *C. rodentium* (Collins et al., 2014; Mullineaux‐Sanders et al., 2019). Mice lacking NLRP3, caspase‐1, IL‐18 and IL‐1β or NLRC4 exhibit exaggerated colonic pathology and an increased bacterial burden in response to *C. rodentium* infection (Liu et al., 2012; Nordlander, Pott, & Maloy, 2013). Interestingly, bone‐marrow transplant experiments revealed that NLRP3 and NLRC4 activity in non‐myeloid cells is sufficient for protection against *C. rodentium* infection (Liu et al., 2012; Nordlander et al., 2013; Song‐Zhao et al., 2013). NLRP6, highly expressed in the colonic epithelial layer, is a major regulator of mucin secretion together with ASC and caspase‐1, and also offers protection against *C. rodentium* (Wlodarska et al., 2014).

Conversely, A/E pathogens encode T3SS effectors which specifically target inflammasome components. NleA (also named EspI), interacts with the pyrin and leucine‐rich repeat domains of ubiquitylated NLRP3, impairing its deubiquitylation and effectively inhibiting recruitment of caspase‐1 to the inflammasome (Yen, Sugimoto, & Tobe, 2015). Importantly, deletion of the gene encoding NleA/EspI results in severe attenuation of *C. rodentium in vivo* (Mundy et al., 2004). The T3SS effector NleF inhibits caspase‐4, as well as caspase‐8 and 9 (Blasche et al., 2013; Pallett et al., 2017). Inhibition of caspase‐4 by NleF blocks secretion of processed IL‐18 following infection of Caco‐2 human IEC‐like cells with EPEC at 4 days post‐infection with *C. rodentium* in mice, which results in reduced neutrophil recruitment (Pallett et al., 2017) (Table 1). Notably, inflammasome responses to the colonic, non‐invasive, pathogen *C. rodentium*, differ significantly from those triggered by *Salmonella*, an invasive pathogen that causes inflammation of the small intestine, as discussed next.

## 5 THE INFLAMMASOME AND *SALMONELLA* INFECTION

*Salmonella enterica subsp. enterica* is divided into typhoidal (e.g. *S*. Typhi) and non‐typhoidal (e.g. *S*. Typhimurium) serovars, which cause enteric (typhoid) fever and gastroenteritis, respectively (Johnson, Mylona, & Frankel, 2018). *S. enterica* encodes two T3SSs within pathogenicity islands called SPI‐1 and SPI‐2. The T3SS effectors injected into the host during infection target and manipulate many host pathways, allowing invasion and persistence of *Salmonella* in the host (Jennings, Thurston, & Holden, 2017; Pinaud, Sansonetti, & Phalipon, 2018). In particular, SPI‐1 effectors are essential for the gastrointestinal stage of the infection, generally involved in invasion of IECs and localised inflammation in the small intestine. In contrast, SPI‐2 effectors are required for the systemic spread of the infection, as they allow survival and replication of the bacteria inside host cells (McGhie, Brawn, Hume, Humphreys, & Koronakis, 2009). *Salmonella* therefore encounters intestinal myeloid cells, such as microfold cells (M cells), DCs and macrophages, and neutrophils and macrophages at other systemic sites.

The interaction of *S*. Typhimurium with inflammasomes *in vitro* using murine bone marrow‐derived macrophages has led to important discoveries, such as the specific and rapid activation of NLRC4 by SPI‐1‐expressing *Salmonella* (Mariathasan et al., 2004). We briefly summarise a large body of work in this area and refer readers to recent reviews (Crowley, Knodler, & Vallance, 2016; Wemyss & Pearson, 2019). In mouse macrophages *S*. Typhimurium flagellins and SPI‐1 T3SS needle and rod are strong activators of NAIP‐NLRC4 inflammasome (Franchi et al., 2012; Yang et al., 2013); reduced SPI‐1 T3SS expression results in both late and weak activation of NLRP3 and NLRC4. NLRP3 is activated through the detection of cytosolic LPS by caspase‐4/11 (Broz et al., 2012; Knodler et al., 2014) and through interferon‐inducible guanylate‐binding proteins (GBPs) (Shenoy et al., 2012; Meunier et al., 2014; Pilla et al., 2014; Santos et al., 2018). The mechanism for canonical NLRP3 activation during *S*. Typhimurium infection has yet to be fully elucidated, although reduced glycolytic flux caused by infection in macrophages has been proposed as a trigger (Sanman et al., 2016). In agreement with their dual roles, both NLRC4 and NLRP3 are recruited to inflammasome foci, along with caspase‐1 and caspase‐8 (Broz, Newton, et al., 2010; Man et al., 2013; Man et al., 2014; Qu et al., 2016; Bierschenk et al., 2019) (Figure 2b). Additionally, CARD9, an adaptor protein involved in immune signalling, inhibits pro‐IL‐1β expression and NLRP3 inflammasomes during *S*. Typhimurium infection. CARD9 acts via the spleen tyrosine kinase, which blocks ASC speck formation and caspase‐8 recruitment to inflammasome foci (Pereira, Tourlomousis, Wright, Monie, & Bryant, 2016). Macrophages are a heterogeneous cell type and tissue‐resident macrophages differ from the bone marrow‐derived macrophages often used for *in vitro* studies. Indeed, intestinal mononuclear phagocytes can stay unresponsive to commensal bacteria but activate the NLRC4 inflammasome in response to *Salmonella* and trigger neutrophil recruitment (Franchi et al., 2012).

As compared to mouse macrophages, the response of human macrophages to *Salmonella* infection is poorly understood. While human NAIP‐NLRC4 can detect purified *S*. Typhimurium needle, rod and flagellin components, the importance of NAIP during infection is less well‐defined (Kortmann et al., 2015, Reyes Ruiz et al., 2017). Infection of monocytes with *S*. Typhimurium activates the NLRP3 inflammasome (Diamond et al., 2017), whereas in macrophages both NLRP3 and NLRC4 are activated (Yang et al., 2013; Baker et al., 2015; Bierschenk et al., 2019). Although *S*. Typhimurium does not activate caspase‐4‐dependent pyroptosis in naive macrophages, in IFNγ‐activated macrophages GBP1 recruits caspase‐4 directly to *S*. Typhimurium leading to its activation and enhanced pyroptosis (Fisch et al., 2019). *S. enterica* serovar Typhi can activate inflammasomes in a flagellin‐dependent manner in human primary monocyte‐derived macrophages (Kortmann et al., 2015), while in the monocytic THP‐1 cell line, which expresses lower levels of *NAIP* (Kortmann et al., 2015), IL‐1β release in response to *S*. Typhi infection is flagellin‐independent and caspase‐dependent (Winter et al., 2015).

The role of mouse inflammasomes in the defence against *S*. Typhimurium *in vivo* has been extensively studied, which we discuss here briefly. *Salmonella* invades and replicates within non‐phagocytic cells such as IECs and triggers local inflammation and epithelial barrier breakdown. Inflammasomes are also activated within IECs infected with *Salmonella*. The NAIP‐NLRC4 inflammasome and caspase‐11 promote cell death and expulsion of infected IECs from the intestinal mucosa, which reduces the available replicative niches for *S*. Typhimurium (Knodler et al., 2010; Knodler et al., 2014; Sellin et al., 2014; Rauch et al., 2017). However, during later stages of infection, *Salmonella* dampen NAIP‐NLRC4 inflammasome activation by reducing SPI‐1 expression and up‐regulating the more immunologically silent SPI‐2 T3SS (Miao, Mao, et al., 2010; Zhao et al., 2016; Pérez‐Morales et al., 2017; Reyes Ruiz et al., 2017) and down‐regulating flagellin expression (Cummings, Wilkerson, Bergsbaken, & Cookson, 2006; Ilyas et al., 2018). Notably, NLRC4‐driven pyroptosis of macrophages infected with *S*. Typhimurium results in bacterial liberation *in vivo* and subsequent phagocytosis and killing by neutrophils, which are resistant to NLRC4‐mediated pyroptosis (Miao, Leaf, et al., 2010; Chen et al., 2014). Loss of *Casp1* increases susceptibility of mice to *S*. Typhimurium (Lara‐Tejero et al., 2006; Raupach, Peuschel, Monack, & Zychlinsky, 2006). In addition to reduced IL‐1β and IL‐18 that are required for an optimal Th1 immune response for bacterial clearance, the loss of *Casp1* also reduces bacterial uptake by neutrophils. As a result, in *Casp1* ^*−/−*^ mice macrophage pyroptosis through caspase‐11 leads to higher extracellular bacterial load, resulting in greater susceptibility to infection (Broz et al., 2012). *Casp1/Casp11‐*double knockout mice, whose cells are resistant to pyroptotic lysis, have fewer extracellular bacteria and are therefore less susceptible than *Casp1* ^*−/−*^ single knockouts (Broz et al., 2012). These findings point towards a role of inflammasomes and caspase‐1/11 in pyroptosis, neutrophil function and adaptive immune responses during *S*. Typhimurium infection *in vivo*. Furthermore, the effector SlrP (*Salmonella* leucine‐rich repeat protein), secreted through both T3SSs, inhibits inflammasome activation and IL‐1β release in the small intestine; higher IL‐1β levels during infection with a Δ*slrP* strain promote anorexia and increases disease severity (Rao et al., 2017) (Table 1).

Additionally, AIM2 has been reported to have a role in preserving epithelial integrity of the intestinal barrier during *Salmonella* infection, but it is not clear whether this defence mechanism involves inflammasome or non‐inflammasome‐dependent functions of AIM2 (Hu et al., 2016). Conversely, NLRP12 plays an undefined role in dampening the immune response to *S*. Typhimurium, and deficiency of this NLR leads to resistance to infection (Vladimer et al., 2012; Zaki et al., 2014), independently of caspase‐1 (Zaki et al., 2014). In summary, tissue‐ and cell type‐specific inflammasome signalling is protective against *Salmonella* infection.

## 6 *SHIGELLA* – INFLAMMASOME INTERACTIONS ARE CELL‐TYPE SPECIFIC

Infection by *Shigella* is a leading cause of diarrhoea in children in low‐ and middle‐income countries, where it is associated with high mortality rates (Tickell et al., 2017). There are four *Shigella* species, two of which (*S. flexneri* and *S. sonnei*) cause 90% of all infections. *Shigella* infection can cause shigellosis or bacterial dysentery, a severe diarrhoea containing blood and/or mucous which can be quickly spread in the population due to its low infective dose (Shane et al., 2017). The vast majority of research has been conducted on the most prevalent species, *S. flexneri*, which relies on its ability to utilise a T3SS to invade and replicate within the intestinal mucosal epithelium, causing inflammation and cell death. Adult mice are colonised poorly by *Shigella in vivo*; however the zebrafish model of infection has been useful in better dissecting host–pathogen interactions (Mostowy et al., 2013). In both epithelial cells and macrophages *S. flexneri* rapidly escapes the endocytic vacuole and accesses the cytosol where it is vulnerable to cellular defence mechanisms. Despite the cytosolic localisation of *S. flexneri* in both cell types, the outcome of infection is different; in macrophages cytosolic *S. flexneri* activates inflammasomes and induces rapid pyroptosis (Hilbi et al., 1998), whereas in epithelial cells *S. flexneri* avoids inflammasome activation and pyroptosis and instead triggers a delayed calpain‐dependent necrotic cell death mediated by the effector VirA (Bergounioux et al., 2012).

Compared to studies with *Salmonella*, much less is currently known about the roles inflammasomes play during *Shigella* infection. *S. flexneri* LPS can be detected by caspase‐4 *in vitro* and in epithelial cells (Kobayashi et al., 2013; Shi et al., 2014) (Figure 2c; Table 1). To counteract this and prolong epithelial cell survival, *S. flexneri* delivers the T3SS effector OspC3. OspC3 interacts with the cleaved caspase‐4 subunit p19 and inhibits its activation by preventing heterodimerisation of the caspase‐4 p19 and p10 subunits (Kobayashi et al., 2013). The importance of OspC3 was confirmed in a synthetic ‘bottom‐up’ approach to identify effectors that inhibit epithelial cell death. This study also identified the effectors OspD2 and IpaH1.4 (Mou, Souter, Du, Reeves, & Lesser, 2018). OspD2 controls the amount of VirA translocated into the host cell, thus regulating VirA‐mediated necrosis (Mou et al., 2018). IpaH1.4 has previously been demonstrated to suppress NF‐κB signalling by acting as an E3 ubiquitin ligase that ubiquitinates and degrades HOIP (HOIL‐1 interacting protein), a component of the linear ubiquitin chain assembly complex (de Jong, Liu, Chen, & Alto, 2016).

In macrophages, *S. flexneri* activates a number of inflammasome pathways leading to rapid cell death. The T3SS needle and rod proteins (MxiH and MxiI) are recognised by human NAIP (and mouse Naip1 and 2 respectively) to induce NLRC4 inflammasome activation (Kofoed & Vance, 2011; Yang et al., 2013; Suzuki, Franchi, et al., 2014; Zhao et al., 2016). Cellular damage resulting from pore formation by the T3SS effector IpaB may also contribute to NLRC4 inflammasome activation (Senerovic et al., 2012). In addition, IpaH7.8 promotes NLRC4 and NLRP3 inflammasome activation by ubiquitinating glomulin (GLMN) and eliciting its degradation (Suzuki, Mimuro, et al., 2014). GLMN is an inflammasome repressor which specifically targets cellular inhibitor of apoptosis proteins 1 and 2 (cIAP1 and cIAP2), members of the inhibitor of apoptosis family of RING‐E3 ligases, resulting in reduced cIAP E3 ligase activity and consequently diminished cIAP‐mediated inflammasome activation. IpaH7.8‐mediated GLMN depletion therefore results in increased inflammasome‐mediated death of macrophages (Suzuki, Suzuki, Mimuro, Mizushima, & Sasakawa, 2018). IpaH7.8 can also activate murine NLRP1B through ubiquitination and functional degradation of NLRP1B, whereby degradation of the amino‐terminal of NLRP1B releases a carboxyl terminal fragment able to activate caspase‐1 (Neiman‐Zenevich, Stuart, Abdel‐Nour, Girardin, & Mogridge, 2017; Sandstrom et al., 2019). Interestingly, IpaH7.8 does not activate human NLRP1 and, therefore, this pathway would not contribute to inflammasome activation during human *Shigella* infection. This may play a role in the human specificity displayed by *S. flexneri*, which does not naturally infect mice (Sandstrom et al., 2019) (Figure 2c). Conversely, during proliferation within epithelial cells, *S. flexneri* produces LPS with hypoacylated lipid A which, when used to stimulate macrophages prior to *S. flexneri* infection, results in reduced caspase‐1 activation and IL‐1β production (Paciello et al., 2013). Such LPS modification may also serve to reduce inflammasome activation in macrophages *in vivo*. Accordingly, *S. flexneri* employs diverse strategies to fine‐tune inflammasome activation.

Similarly to *S. flexneri*, *S. sonnei* also induces caspase‐1 dependent pyroptosis of macrophages. However, the level of cell death induced by *S. sonnei* is lower, as internalisation and vacuole escape are less efficient, resulting in fewer cytosolic bacteria. This is due to shielding of the T3SS by the O‐antigen, which is present on the lipid A‐core as LPS and as a group 4 capsule (Watson et al., 2019). The inflammatory environment caused by *Shigella*‐induced macrophage pyroptosis recruits neutrophils to the infection site. While this initially aids infection by destabilising the epithelial barrier (Perdomo, Gounon, & Sansonetti, 1994), it ultimately leads to bacterial killing and the resolution of infection. Interestingly, recent evidence suggests the O‐antigen of *S. sonnei* enables the bacteria to resist neutrophil killing in zebrafish (Torraca et al., 2019), highlighting the need for further work to characterise this species. Altogether, the presence of effectors and strategies that help evade inflammasomes in *Shigella* spp. suggests that the host deploys inflammasome pathways for self‐defence.

## 7 *L. MONOCYTOGENES* BENEFITS FROM INFLAMMASOME ACTIVATION

*L. monocytogenes* is a Gram‐positive food‐borne pathogen (Allerberger & Wagner, 2010). Seminal work from Pascale Cossart's lab and others implicated two proteins in the virulence of *L. monocytogenes*: listeriolysin O (LLO; encoded by the hlyA gene), a cholesterol‐dependent pore‐forming haemolysin, and ActA, a surface bacterial protein that mediates actin‐based motility within host cells and cell‐to‐cell spreading of the bacterium (Hamon, Ribet, Stavru, & Cossart, 2012; Pizarro‐Cerdá, Kühbacher, & Cossart, 2012). After invasion of IECs and myeloid cells, *Listeria* spreads to deeper tissues such as the liver and spleen, and can also cross the blood–brain barrier and the placenta in pregnant women. Although *Listeria* resides and replicates in the cytosol of different cell types, its interactions with inflammasomes are best understood in macrophages.

Upon infection of mouse or human macrophages, *L. monocytogenes* can be detected by multiple inflammasomes, including AIM2, NLRC4, NLRP3, NLRP6, caspase‐11 and NLRP1B. On the bacterial side, LLO (Meixenberger et al., 2010; Hamon & Cossart, 2011; Eldridge, Sanchez‐Garrido, Hoben, Goddard, & Shenoy, 2017), bacterial RNA (Kanneganti et al., 2006), bacterial DNA (Sauer et al., 2010) and the secreted protein p60 (Schmidt & Lenz, 2012) have been implicated in inflammasome activation (Table 1). Notably, conflicting findings have occasionally been reported, which may have arisen from the use of different *L. monocytogenes* strains, cell‐types (monocytes vs. macrophages), species (human vs. mouse) and/or pre‐treatment with TLR ligands (e.g. LPS) or type I or type II interferons (IFNα/β or IFNγ respectively). Here we summarise key findings on inflammasome activation by *L. monocytogenes* and refer readers to a detailed discussion elsewhere (Theisen & Sauer, 2016).

In mouse macrophages, AIM2 is the major inflammasome sensor of *L. monocytogenes* (Sauer et al., 2010; Warren et al., 2010; Wu, Fernandes‐Alnemri, & Alnemri, 2010). The commonly used *L. monocytogenes* strain 10403S undergoes occasional lysis in the cytosol, leading to the release of its DNA and direct activation of the AIM2 inflammasome. A mutant lacking the *lmo2473* gene lyses more in the cytosol of infected macrophages and hyperactivates the AIM2‐ASC‐caspase‐1 inflammasome pathway (Sauer et al., 2010). AIM2 expression requires type I IFN signalling, consistent with which, *L. monocytogenes*‐induced inflammasome activation is severely reduced in type I IFN‐receptor deficient (*Ifnar* ^*−/−*^) macrophages. *L. monocytogenes* is a poor activator of NLRC4 (Sauer et al., 2010; Warren et al., 2010; Sauer et al., 2011), but can activate NLRP6, caspase‐11 (Hara et al., 2018) and NLRP1B (Neiman‐Zenevich et al., 2017) in mouse macrophages, and NLRP3 in mouse bone‐marrow derived DCs (Clark, Schmidt, McDermott, & Lenz, 2018). Most *L. monocytogenes* strains turn down flagellin expression at 37°C and thus evade detection by TLR5 and the NAIP5‐NLRC4 pathway (Theisen & Sauer, 2016). However, due to a mutation in MogR (*lmo0674*), 10403S exhibits low basal flagellin expression which weakly activates the NLRC4 inflammasome (Gründling, Burrack, Bouwer, & Higgins, 2004). *L. monocytogenes* engineered to overexpress flagellins results in severe attenuation *in vivo* in an NLRC4‐dependent manner and these strains were also found to be poor vaccine candidates (Sauer et al., 2011). Surprisingly, bacterial lipoteichoic acid activates NLRP6 and caspase‐11 in mouse macrophages infected with *L. monocytogenes* strain EGD (Hara et al., 2018) (Table 1). NLRP6‐ASC complexes recruited both caspase‐1 and caspase‐11, where the role of active caspase‐11 was to promote caspase‐1 activation, which in turn processed IL‐18 and IL‐1β cytokines (Figure 2d). Importantly, in human cells, NLRP6‐silencing does not affect caspase‐1 activation by *L. monocytogenes* EGD (Meixenberger et al., 2010), which points towards species‐specific differences. Furthermore, genome sequencing studies performed by P. Cossart have confirmed that the EGD strain is markedly different from 10403S and EGDe strains and has a mutation in the master transcriptional regulator PrfA (PrfA*) which results in constitutive expression of various virulence genes (Bécavin et al., 2014). It is plausible that these differences also contribute to strain‐specific responses in host cells.

Studies in human macrophages showed that NLRP3 is involved in detection of *L. monocytogenes*; however, this is context‐dependent and has been observed in some (Kanneganti et al., 2006; Meixenberger et al., 2010; Wu et al., 2010; Shenoy et al., 2012; Fernandes‐Alnemri et al., 2013), but not other (Franchi, Kanneganti, Dubyak, & Núñez, 2007; Sauer et al., 2010; Warren et al., 2010), studies. While silencing of NLRP3 in human peripheral blood mononuclear cells reduces inflammasome activation by *L. monocytogenes* strain EGD, silencing of NLRP1, NLRC4, NLRP12, AIM2, RIP2 or NOD2 has no effect, ruling out their involvement (Meixenberger et al., 2010). *Listeria*‐mediated NLRP3 activation is mediated by LLO (Meixenberger et al., 2010; Eldridge et al., 2017) (Table 1). Moreover, recombinant LLO can trigger potassium efflux (Hamon & Cossart, 2011), suggesting that extracellular LLO may also trigger NLRP3 activation. Mechanistically, NLRP3 activation during *Listeria* infection requires lysosomal acidification, rupture and the release of cathepsin B (Meixenberger et al., 2010). Infection of IFNγ‐primed human THP1 cells with *L. monocytogenes* strain 10403S results in enhanced NLRP3 activation assisted by the IFNγ‐inducible GBP5 (Shenoy et al., 2012). Altogether, these studies are consistent with the recently identified AIM2‐independent, NLRP3‐dependent detection of cytosolic DNA in human, but not mouse, myeloid cells (Gaidt et al., 2017). This new DNA‐sensing pathway in human cells also requires lysosomal damage (Gaidt et al., 2017). Therefore, *L. monocytogenes*, plausibly via both LLO and release of DNA in the cytosol, activates the human NLRP3 inflammasome and the murine AIM2 inflammasome (Figure 2d).

Early *in vivo* studies on *L. monocytogenes* pointed towards a protective role of inflammasome‐related genes (Hirsch, Irikura, Paul, & Hirsh, 1996; Labow et al., 1997; Tsuji et al., 2004); however, these studies were carried out in mice with a mixed C57BL/6 and 129/S background which also carry an inactivating passenger mutation in *Casp11*. More recent studies suggest that loss of *Il18*, *Nlrp3* or *Asc* increases resistance to *L. monocytogenes*, and loss of *Casp1/11* ^*−/−*^ does not markedly affect survival or adaptive immune responses (Lochner et al., 2008; Sauer et al., 2011; Tsuchiya et al., 2014; Clark et al., 2018). Intriguingly, *Nlrp6* ^*−/−*^ and *Casp11* ^*−/−*^ mice, which would not recognise *Listeria* LTA, are also more resistant to *L. monocytogenes* infection (Hara et al., 2018). However, increased resistance of *Nlrp6* ^*−/−*^ mice to *L. monocytogenes* has also been attributed to increased canonical NF‐κB signalling and myeloid cell responses that better restrict bacterial growth (Anand et al., 2012). As inflammasome‐driven inflammation increases neutrophil influx, these findings suggest that a heightened neutrophil‐response is detrimental in the defence against *L. monocytogenes in vivo*. Taken together, most *in vivo* studies in mice suggest that inflammasome activation benefits *Listeria* and is detrimental to the host.

## 8 *HELICOBACTER PYLORI,* INFLAMMASOME STIMULATION AND PERSISTENT INFECTION

*H. pylori* is found in the gastric mucosa of around 50% of the world population but only a minority of infected individuals will develop long‐term clinical symptoms (Abadi & Kusters, 2016). Individuals with an active chronic infection can develop gastric ulcers, chronic gastritis, gastric mucosa‐associated lymphoid tissue (MALT) lymphoma and gastric cancers (Chey et al., 2017). Inflammasome‐mediated recognition of *H. pylori* in myeloid cells is mainly attributed to NLRP3 through a not well‐defined mechanism requiring expression of the bacterial vacuolating cytotoxin A (VacA) and the cytotoxin‐associated genes pathogenicity island, which encodes the cytotoxin associated gene A (CagA) and the type IV secretion system (T4SS) (Kim, Park, Franchi, Backert, & Núñez, 2013; Semper et al., 2014; Kameoka et al., 2016). It is tempting to speculate that VacA, which forms pores in the plasma membrane, causes mitochondrial damage, increases ROS levels and induces cell apoptosis, may likewise contribute to NLRP3 activation. Caspase‐1, IL‐1β and IL‐18 play an important role in *H. pylori* pathogenesis *in vivo*, but whether inflammasome activation benefits the host or the pathogen remains to be defined (Hitzler et al., 2012; Kim et al., 2013). The role of NLRP3 in promoting (Hitzler et al., 2012; Koch et al., 2015; Arnold et al., 2017) versus restricting (Semper et al., 2014) *H. pylori* infection also remains unclear. These differences may be due to the use of mice of different ages, differences in the microbiota or the *H. pylori* strains, or to variations in the methodologies used in the month‐long infection model. However, supporting the role of inflammasomes in promoting *H. pylori* pathogenesis, the protective role of Mucin1 expression in the gastric mucosa has been linked to the down‐regulation of NLRP3 expression and consequent IL‐1β release (Ng et al., 2016). Moreover, the NLRC4 inflammasome is activated during *H. pylori* infection of gastric epithelial cells in a T4SS‐dependent manner, leading to IL‐18 release and robust neutrophil recruitment. NLRC4 activation promotes *H. pylori* survival as the resulting IL‐18 dampens the IL‐17 response, reducing antimicrobial peptide production and favouring tissue damage and inflammation (Semper, Vieth, Gerhard, & Mejías‐Luque, 2019) (Figure 2e). While inflammasome activation facilitates *H. pylori* infection, exacerbated inflammation and loss of the replicative niche may prove to be detrimental for both the host and the pathogen. Thus, it is not surprising that *H. pylori* also employs immune evasion strategies that include the expression of a modified LPS that is less pyrogenic than that of other Gram‐negative enteric pathogens (Moran, 2007) and a distinct flagellin that fails to activate TLR5 and the NLRC4 inflammasome (Andersen‐Nissen et al., 2005; Matusiak et al., 2015). In summary, inflammasome activation in response to *H. pylori* infection is detrimental for the host, as it promotes *H. pylori* survival in the gastric mucosa.

## 9 *YERSINIA* SPP. AND INFLAMMASOME MANIPULATION

*Yersinia enterocolitica* and *Yersinia pseudotuberculosis* are food‐borne Gram‐negative enteric pathogens that cause a self‐limiting enterocolitis, often presented as terminal ileitis in humans, but can also lead to invasive disease and septicaemia (Galindo, Rosenzweig, Kirtley, & Chopra, 2011). *Yersinia* spp. invade and survive in IECs (Ligeon et al., 2014; Valencia Lopez et al., 2019), cross the intestinal epithelium and target mesenteric lymph nodes (Galindo et al., 2011). Inflammasome activation and evasion by *Yersinia* spp. is best studied in murine macrophages. The NLRP3 and NLRC4 inflammasomes are activated by the detection of bacterial T3SS and the translocation of the pore‐forming translocon components *Yersinia* outer protein B (YopB) and YopD (Brodsky et al., 2010; Zwack et al., 2015). The PYRIN inflammasome is activated through the RhoA GTPase‐inhibiting activity of YopE and YopT (Chung et al., 2016; Ratner et al., 2016) (Figure 2f). To counteract detection by the inflammasomes, *Yersinia* encodes the effector YopK, which interacts with the T3SS and regulates translocation of effectors (Brodsky et al., 2010; Zwack et al., 2015), and YopM, a homologue of the *Salmonella* SlrP which inhibits caspase‐1 (LaRock & Cookson, 2012) and manipulates host kinases to inhibit PYRIN (Chung et al., 2016; Ratner et al., 2016) (Table 1). Both YopK and YopM are thus essential for *Yersinia* virulence. Furthermore, during infection with *Y. enterocolitica*, β1‐integrin signalling triggered by the adhesin invasin increases pro‐IL‐18 levels in IECs; notably, these outcomes are counteracted by YopH and YopE (Thinwa, Segovia, Bose, & Dube, 2014).

The acetyltransferase YopJ (YopP in *Y. enterocolitica*) is a homologue of *Salmonella* AvrA that inhibits transforming growth factor beta‐activated kinase 1 (TAK1), IκB kinase β (IKKβ) and mitogen‐activated protein kinase (MAPK) kinases, inhibiting pro‐inflammatory signalling in response to TLR and/or TNF signalling (Peterson et al., 2016; Pinaud et al., 2018). TAK1 inhibition promotes non‐canonical RIPK1‐FADD‐caspase‐8‐dependent GSDMD cleavage, pore formation and cell death in mouse macrophages. The resulting potassium efflux triggers NLRP3 and caspase‐1 activation, and release of IL‐1β (Philip et al., 2014; Orning et al., 2018) (Figure 2f). In addition, blockade of TNF‐mediated pro‐survival NF‐κB and MAPK signalling by YopJ can also lead to RIPK1‐dependent apoptosis (Weng et al., 2014; Peterson et al., 2016; Peterson et al., 2017). Further, YopJ‐mediated inhibition of the Nod2 pathway has also been linked to Nod2‐dependent activation of caspase‐1 and IL‐1β release from Peyer's patches, and loss of intestinal barrier function in mice (Meinzer et al., 2012). During *Y. pseudotuberculosis* infection *in vivo*, YopJ‐driven cell death pathways promote bacterial clearance and host survival, seemingly counteracting the pathogen‐mediated blockade of inflammatory signalling (Meinzer et al., 2012; Philip et al., 2014; Peterson et al., 2017).

In addition to secreted effectors, *Yersinia* LPS can also activate inflammasomes. However, *Yersinia* evades detection by mouse caspase‐11 *in vivo* by deacylating its lipid A to four lipid chains at 37°C (Hagar, Powell, Aachoui, Ernst, & Miao, 2013). It is possible that human caspase‐4, which can recognise tetra‐acylated LPS from *Francisella novicida,* might also recognise tetra‐acylated *Yersinia* lipid A (Lagrange et al., 2018). While its role during infection with enteric *Yersinia* species *in vivo* is yet to be defined, mouse NLRP12, whose activation mechanisms remain poorly understood, also contributes to IL‐1β release from bone marrow‐derived macrophages infected with *Y. pseudotuberculosis* or *Y. enterocolitica* (Vladimer et al., 2012). Taken together, inflammasome activation during *Yersinia* infection is beneficial for the host as it promotes bacterial clearance (Brodsky et al., 2010; Meinzer et al., 2012; Philip et al., 2014; Peterson et al., 2017).

## 10 CONCLUSIONS

Inflammasomes are key soluble cytosolic surveillance devices that recognise and respond to multiple virulence determinants of both Gram‐negative and Gram‐positive enteric pathogens. Moreover, they operate in multiple cell types that come into early contact with enteric pathogens. Activation of the inflammasomes can trigger the release of pro‐inflammatory cytokines or expel host cells containing intracellular pathogens via cell death. For this reason, it is not surprising that pathogenic bacteria have acquired survival mechanisms to mitigate these host responses. On the other hand, an exuberant inflammasome‐driven inflammatory response results in heightened neutrophil influx and tissue damage, which can promote the systemic spread of invasive pathogens. The pathogens covered in this review selectively infect the gastric mucosa (*Helicobacter*), invade through the small intestine (*Salmonella*, *Listeria, Yersinia*) or colon (*Shigella*), or are restricted to the colonic mucosa (*C. rodentium*). Multiple tissues can be colonised upon invasion, including the lymph nodes, blood, spleen, liver, brain or the foetus, and localised cell‐type specific inflammasome responses remain to be better understood. Although the causal role of caspase‐4 and pyroptosis in murine endotoxic shock is well established, more work is required to clarify the role of inflammasomes in infectious sepsis in humans. Future work should also explore whether immunomodulation of inflammasomes with small molecules (e.g. MCC950) could promote microbial clearance by reducing deregulated inflammation.

Pascale Cossart was among the pioneers who championed studies on the interactions of intracellular bacteria with various host cell machineries, including membrane‐bound surface receptors activated during bacterial entry, manipulation of the cytoskeletal and endocytic trafficking systems (Cossart, Boquet, Normark, & Rappuoli, 1996). Studies from her group and others also ushered the phase of discovery of cytosolic immune surveillance pathways aided by the use of various bacterial pathogens as invaluable cell biological tools. Studies have now revealed the benefits and disadvantages of inflammasome signalling in host defence. No doubt, the near future will reveal yet new inflammasome functions and other subversion mechanisms, writing new a chapter of the never‐ending story of an intimate relationship between bacteria and the host.

## CONFLICT OF INTEREST

The authors have no interests to declare.

## AUTHOR CONTRIBUTIONS

J.S.G., S.L.S., A.C., A.R.S. and G.F. wrote the review. J.S.G. and A.R.S. created the figures.
